# Delayed Diagnosis of Coarctation of the Aorta as a Cause of Secondary Hypertension in an Adult Female With Congenital Heart Disease: A Missed Opportunity for an Earlier Diagnosis?

**DOI:** 10.7759/cureus.91695

**Published:** 2025-09-05

**Authors:** Kamran D Ahmad, Ellie Anderson, Zahid Khan, Shah R Mohdnazri

**Affiliations:** 1 Cardiology, Mid and South Essex NHS Foundation Trust, Southend-on-Sea, GBR; 2 Cardiology, Southend University Hospital, Southend-on-Sea, GBR; 3 Cardiology, University of South Wales, Pontypridd, GBR; 4 Cardiology, University of Buckingham, London, GBR; 5 Cardiology, Barts Heart Centre, London, GBR; 6 Cardiology, Mid and South Essex NHS Foundation Trust, Southend University Hospital, Southend-on-Sea, GBR

**Keywords:** bicuspid aortic valve disease, coa: aortic coarctation, coa (coarctation of the aorta), coarctation stent, infective endocarditis, resistant hypertension, severe coarctation of the aorta, severe hypertension, stenting aorta, ventricular septal defect (vsd)

## Abstract

Coarctation of the aorta (CoA) is a narrowing of the aorta that typically occurs just after the point where the left subclavian artery branches off. This is usually around the area where the ductus arteriosus used to connect to the aorta in foetal life. Most cases are sporadic; however, genetic associations have also been established in some cases. Generally, it leads to heart failure symptoms at an early age, but later presentation beyond adulthood often remains asymptomatic. Clinical suspicion about the condition usually arises in patients with uncontrolled blood pressure and reduced or absent femoral pulses. We present a case of a 30-year-old female patient known to have congenital heart disease (ventricular septal defect and moderate tricuspid regurgitation) who suffered from infective endocarditis in her adolescent years (treated successfully medically) and presented years later with uncontrolled blood pressure, palpitations, and headaches. With the help of a workup for hypertension in the young, especially imaging, and a close follow-up, she was diagnosed with CoA, which has a strong association with other congenital heart diseases. She underwent stenting of the lesion, with a successful outcome and reasonable control of blood pressure.

## Introduction

Hypertension can be caused by both primary and secondary factors. Hypertension can also be caused by congenital heart diseases, such as dextro-transposition of the great arteries (D-TGA), bicuspid aortic valve (BAV), tetralogy of Fallot (TOF), syndromic associations such as Turner's and Williams' syndromes, and coarctation of the aorta (CoA) [1-3]. CoA accounts for approximately 4%-6% of all congenital heart diseases, and the incidence is 3-4 cases per 10000 live births [1,3]. Generally, males are more affected than females, with a ratio of 1.27:1 and 1.74:1, respectively [4-6]. It usually presents in childhood, but sometimes, it can be detected late into adulthood, and the symptoms are often nonspecific [6]. Common clues to a suspected diagnosis include headache, epistaxis, cool peripheries, limb claudication, and unequal pulses in the arms. A significant clue is upper-extremity hypertension, which is often resistant in young individuals, justifying the measurement of lower-extremity blood pressure at least once in these patients [6]. Studies have shown that patients with this CoA have a shorter life expectancy and an increased risk of cardiovascular complications; hence, early diagnosis and treatment are crucial [6]. It is worth mentioning that a significant number of asymptomatic adults are not detected until adulthood, resulting in an underestimation of the condition at birth [7].

The clinical presentation of CoA varies depending on the age of presentation, the severity of narrowing, the relationship with the arch vessels, and the formation of collateral vessels [7]. CoA may be suspected in neonates and infants due to right ventricular enlargement associated with decreased left ventricular flow and greater flow through the ductus arteriosus. Infants with patent ductus arteriosus may remain asymptomatic. However, postductal closure, infants with CoA can develop heart failure and/or shock due to acute left ventricular pressure overload. The prevalence of CoA in adults is approximately 10.3% after the age of 40 years, and all these patients generally have upper-extremity hypertension and lower blood pressure in the lower extremities, with delayed femoral pulses [8]. Clinical examination may reveal an ejection systolic murmur at the left upper sternal border radiating to the interscapular area, radio-femoral delay, and hypertension in the upper limbs. We describe an interesting case of CoA, which, among the known congenital cardiac diseases, is probably the best-known entity associated with hypertension and troublesome outcomes if detected later. The patient was treated with CoA stenting, achieving favourable outcomes, and is now living a symptom-free life with routine follow-up care.

## Case presentation

We present the case of a Caucasian female patient in her 30s with palpitations. She was first seen in our cardiology department at the age of 16. She was referred to our clinic at that time during the transition period from paediatric to adult care on a background of infective endocarditis of restrictive peri-membranous ventricular septal defect (VSD) (left-to-right shunt) and moderate tricuspid regurgitation, which was medically managed. The patient was completely asymptomatic, and she was followed up in the outpatient clinic over the following three years with evidence of healed (sterile) vegetation on the tricuspid valve on follow-up echocardiograms. According to the documentation available at that time, she was first noted to have a high clinical blood pressure of 150/90 mmHg at the age of 16. However, there is no evidence of a formal work-up to investigate her for possible causes of hypertension, and she was discharged from the outpatient clinic. According to the available medical records, the patient received only norethisterone tablets. No treatment was considered because of her high clinical BP readings at that time.

She was referred this time for palpitations and associated headaches that occurred on an almost daily basis. She stopped smoking a few years before this visit to our outpatient clinic. She also used progesterone-only oral contraceptives. There was no history of illicit drug use or any other medication. At the clinic, her blood pressure was 140/70 mmHg. Her relevant cardiovascular examination was significant only for a loud pansystolic murmur, heard throughout the precordium but best at the left lower sternal border, consistent with her VSD. A 24-hour ambulatory blood pressure monitor demonstrated a mean BP of 226/119 mmHg (nighttime BP readings were not available). Echocardiography revealed good biventricular systolic function with an ejection fraction of 55%-60%, a small peri-membranous VSD, and a tricuspid aortic valve. The echocardiogram was performed at another centre.

She underwent further investigations to rule out secondary causes of hypertension, which included blood tests such as a full blood count, urea, creatinine, electrolytes, glycated haemoglobin (HbA1c), urine and serum cortisol, insulin-like growth factor-1 (IGF-1), urinary metanephrines, thyroid profile, and renin and aldosterone levels. After blood and urine tests were completed, the patient was advised to start taking amlodipine 5 mg once daily, and one possible reason for this could be that the patient was awaiting a CT renal angiogram to rule out a renal cause for hypertension. The patient was instructed to self-monitor her blood pressure at home.

**Figure 1:**
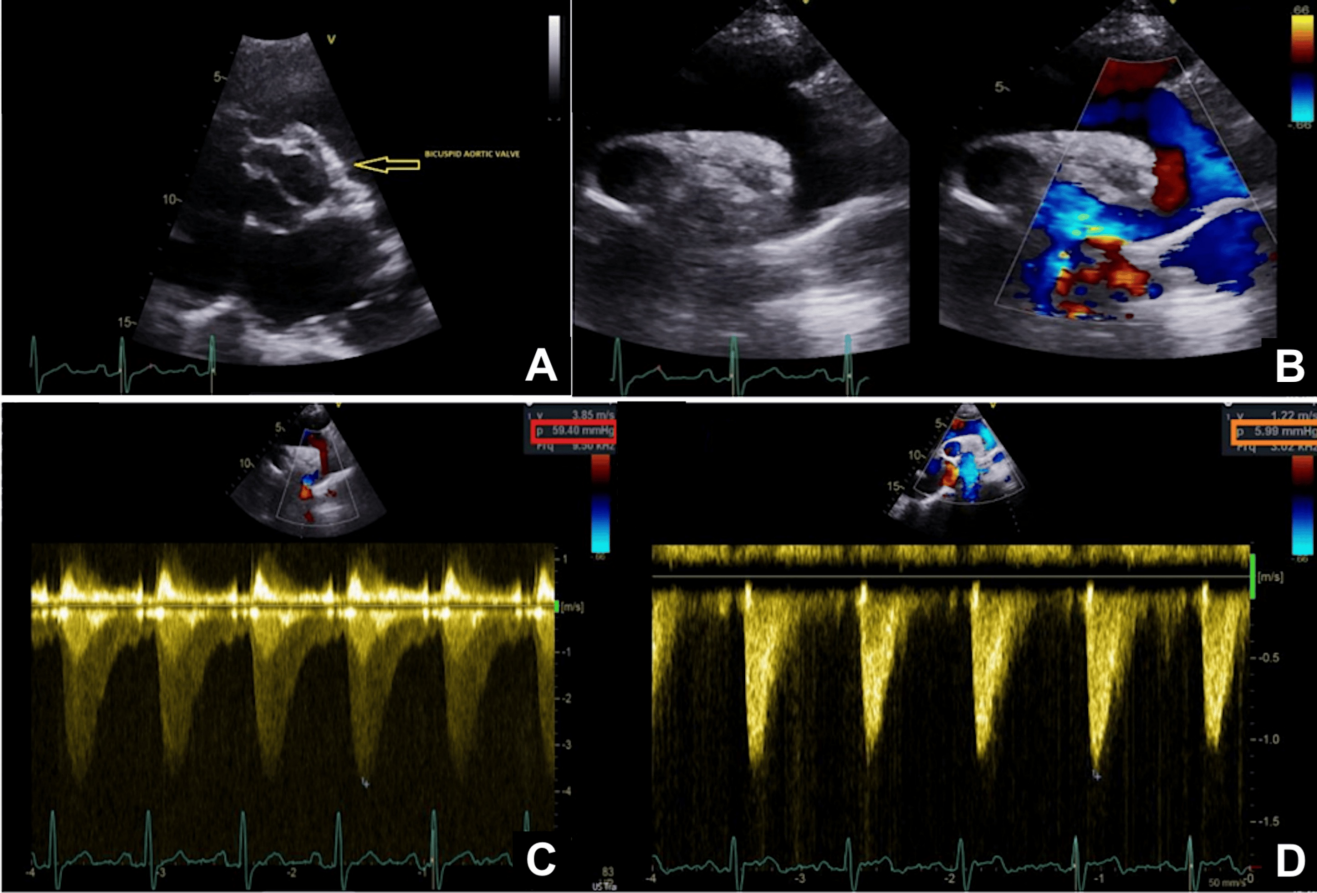
Transthoracic echocardiogram images (A) Bicuspid aortic valve (yellow arrow); (B) normal and colour flow across aortic arch showing turbulence; (C) pre-procedural gradients across coarctation; red box (59.4 mmHg); (D) post-procedural normalised gradients across the same segment; orange box (5.99 mmHg).

**Figure 2:**
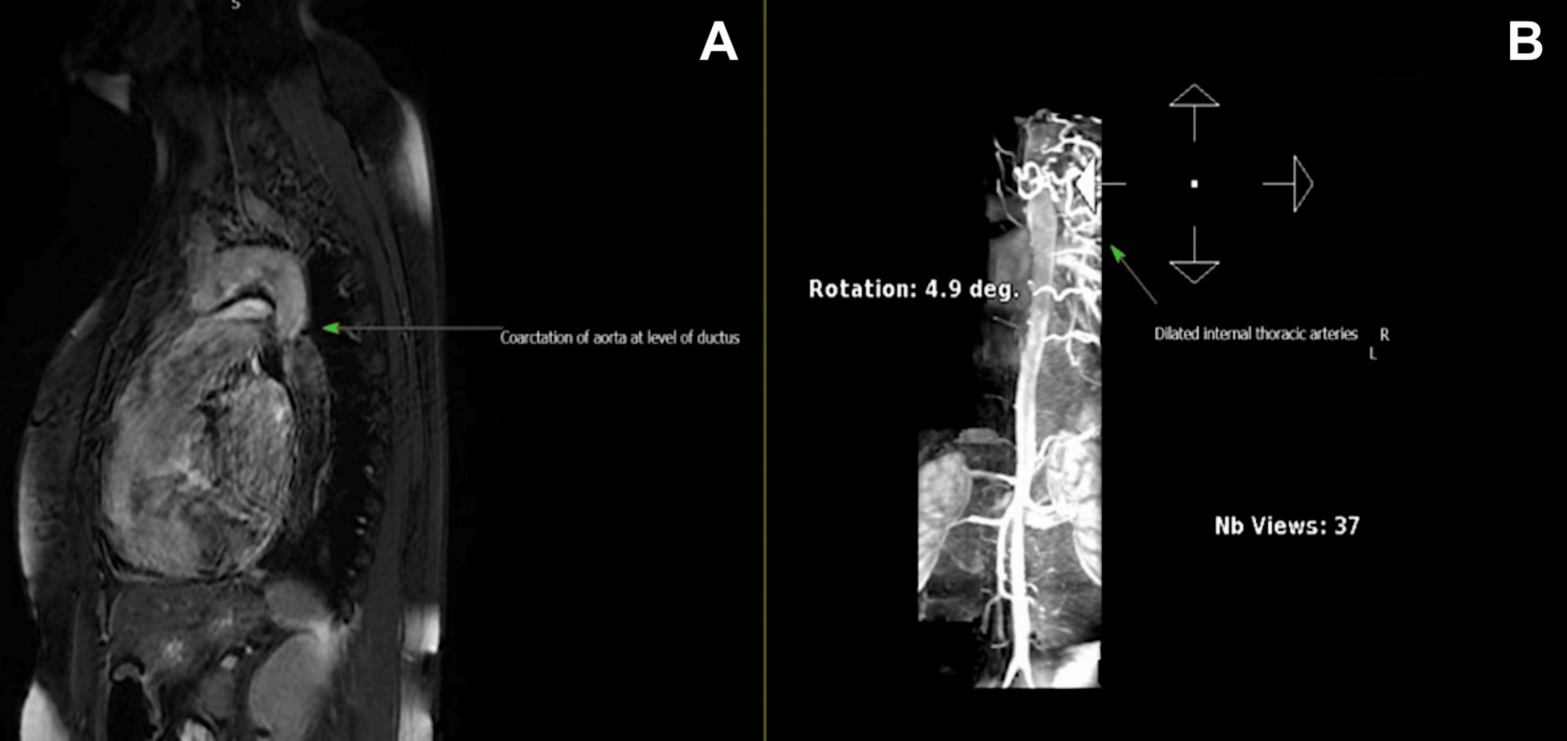
Pre-procedural MRA aorta (A) Coarctation just distal to the ductus; (B) compensatory dilation of the internal thoracic arteries supplying the lower limbs via the inferior epigastric artery.

**Figure 3:**
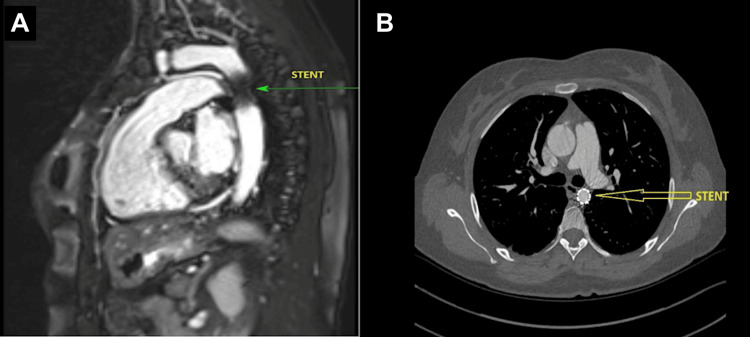
Post-procedure follow-up MRI (A) and CT aortogram (B) showing stent in situ

She underwent magnetic resonance angiography (MRA) of the entire aorta in March 2023, which revealed CoA. Additionally, she was found to have a BAV on both the MRA aorta and the transthoracic echocardiogram performed at our regional tertiary care heart centre (Figures 1-3). A CT coronary angiogram was arranged following this in May 2023, showing severe aortic coarctation in the proximal descending aorta, with prominent intercostal and internal mammary arteries. There was mild calcification of the anterior tricuspid valve, and a small peri-membranous VSD was also observed. The coronary arteries were normal.

The results of blood and urine tests are presented in Table 1.

**Table 1:** Blood and urine test results IGF-1: insulin-like growth factor 1; TSH: thyroid stimulating hormone; HbA1c: glycated haemoglobin

Clinical Tests	Results	Reference Range
Cortisol (0900 hrs)	568	(185–624 nmol/L)
HbA1c	30	(20–42 mmol/mol)
IGF-1	23.7	(9.2–30.4 nmol/L)
24-h urinary cortisol	40	< 200 nmol/day
Urine normetanephrine	0.75	< 3.7 µmol/day
Urine metanephrine	0.29	< 1.3 µmol/day
Urine 3-methoxytyramine	0.39	< 2.6 µmol/day
Plasma aldosterone	170	90–700 pmol/L
Plasma renin	0.5	0.5–3.5 nmol/L/h
Aldosterone: renin ratio	340	< 680 (Conn's unlikely)
TSH	1.8	0.3–5.0 mU/L
Free T4	11.3	7.9–16 pmol/L

Once the diagnosis of CoA and BAV was made, she was also started on bisoprolol 2.5 mg once daily (OD) along with amlodipine 5 mg OD, with reasonable control of blood pressure to as low as 138/78 mmHg. She was initially referred to our regional tertiary cardiac care centre for review at the dedicated Adult Congenital Heart Disease (ACHD) clinic. Following a review in the ACHD clinic, her case was discussed at a dedicated ACHD MDT, with a consensus for her to be treated with percutaneous stenting of the CoA. Subsequently, she was successfully treated at age 32 with stenting of the coarctation (8 Zig/39 mm CP stent of the BiB Balloon) at the centre in May 2023 (Figure 3). After stenting, she was also administered aspirin 75 mg OD for six months.

Following stenting for CoA, the patient was routinely followed up at the ACHD clinic. She had a couple of episodes of dizziness and palpitations four months later. She underwent a gated CT aorta in December 2023 that showed mild mid-stent narrowing, causing a ~30% reduction in calibre between the stent narrowing and the diaphragmatic aorta. No pseudoaneurysm or collaterals were observed, and the aortic root was dilated, with prominence of the posterior aortic cusp. She had been under routine surveillance since then at our regional cardiac centre at the ACHD clinic. Her latest echocardiogram, performed in November 2024, demonstrated normal pressure gradients across the descending thoracic aorta. However, the right ventricle was moderately dilated with good systolic function, and moderate aortic regurgitation was observed across a BAV with an upper normal-sized ascending aorta. She underwent a cardiac MRI scan in October 2024, showing a moderately dilated RV with preserved RV function, moderate TR, a BAV without stenosis and mild AR. She remains clinically stable at age 34 and is still under follow-up.

## Discussion

A vast majority of cases of CoA are sporadic; however, genetic predisposition has also been described, including associations with neurogenic locus notch homolog protein 1 (NOTCH1), multiple C2 domains, transmembrane 2 (MCTP2), and Matrin-3 (MATR3) [6]. CoA is considered a generalised arteriopathy due to its sequelae, such as hypertension-related adverse outcomes, heart failure, intracerebral bleeding, development of premature coronary artery disease, and devastating consequences, such as aortic dissection/aneurysm rupture [6-8]. The exact mechanism of the pathophysiology of CoA is not precise; however, there are a few hypotheses, other than genetic causes, that try to explain the likely mechanism(s), such as abnormal forward intrauterine blood flow hampering the development of the foetal aortic arch and another idea that the PDA tissue abnormally infiltrates into the foetal thoracic aortic wall [9]. CoA is associated with other congenital anomalies of the great vessels and extracardiac complications. In approximately 50%-85% of cases, it is associated with a BAV, and in about 10% of cases, intracranial aneurysms are also encountered [10,11].

According to the latest European Society of Cardiology (ESC) guidelines for the management of adult congenital heart disease, published in 2020, repair of coarctation or re-coarctation is a class 1 indication in the presence of hypertension and an invasively measured peak-to-peak gradient of≥ 20 mmHg, with a preference for catheter-based treatment. Regular follow-up for such patients is mandatory to closely monitor the development of sequelae and complications, especially if they have received an intervention. Cardiac MRI is the preferred modality for this purpose. Generally, three to five yearly imaging surveillance sessions are recommended [12].

Celermajer et al. conducted a single-centre retrospective study in Sydney where they followed up 150 patients with CoA from 1994 to 2013. Of these, 140 (mean age, 35 ± 15 years) underwent surgical repair at the age of five years. They demonstrated an excellent post-intervention survival rate of 98% at 40 and 50 years and 89% at 60 years [13]. Similarly, Luo et al. also described a young man in his 40s who presented with uncontrolled hypertension, chest pain, and deranged kidney function. Despite extensive testing, the diagnosis remains elusive. They performed invasive coronary angiography and aortography to identify significant multivessel disease and CoA. The coronaries were treated with stenting, and the CoA was fixed after stenting for several months. A two-year follow-up showed an improvement in kidney function and controlled hypertension [14].

Ringel et al. published a five-year follow-up for both the COAST (Coarctation of the Aorta Stent Trial) and COAST II (Covered Cheatham-Platinum Stents for Prevention or Treatment of Aortic Wall Injury Associated With Coarctation of the Aorta trial) that showed encouraging results in terms of maintained patency of covered stents up to 60 months post-implant and also a notable reduction in use of antihypertensive medications, although when evaluated for a medium and late follow-up, more reinterventions and complications such as in-stent fractures were encountered [15].

Studies directly comparing the efficacies of surgery and stenting are limited. Yeaw et al. conducted a retrospective analysis of 28 patients from September 2000 to January 2015, noting that stenting was non-inferior to surgical correction in both short- and mid-term follow-up periods [16]. Similarly, Forbes et al. also performed a multi-centre, non-randomised, observational study predominantly in patients aged 6-12 years. They found that patients undergoing stenting or surgery experienced superior haemodynamic and aortic arch imaging improvements compared with those undergoing balloon angioplasty. In contrast, in the short-term and intermediate-term follow-up, stenting patients demonstrated fewer acute complications, albeit higher chances of planned re-intervention [17].

## Conclusions

Our case report highlights the importance of maintaining a high degree of clinical suspicion to screen for secondary hypertension and to be mindful of considering its relatively rare causes, even after performing a minimum standard set of investigations. Clinical examination and awareness of the relationship between different congenital heart diseases and their co-existence, despite initial negative imaging investigations, are invaluable. Early and relevant imaging modalities are vital to arrive at a diagnosis in patients who are already known to have some form of congenital anomaly. This has important clinical implications in terms of long-term adverse outcomes, including early death, CoA if left untreated, and arranging a routine follow-up after intervention.
